# CRISPR/Cas9-Induced Knockout of miR-24 Reduces Cholesterol and Monounsaturated Fatty Acid Content in Primary Goat Mammary Epithelial Cells

**DOI:** 10.3390/foods11142012

**Published:** 2022-07-07

**Authors:** Lian Huang, Jun Luo, Wenchang Gao, Ning Song, Huibin Tian, Lu Zhu, Qianming Jiang, Juan J. Loor

**Affiliations:** 1 Shaanxi Key Laboratory of Molecular Biology for Agriculture, College of Animal Science and Technology, Northwest A&F University, Yangling, Xianyang 712100, China; kin8248806@163.com (L.H.); gaowenchang666@163.com (W.G.); songning@nwafu.edu.cn (N.S.); tianhuibin@nwafu.edu.cn (H.T.); zl411143710@163.com (L.Z.); 2 Qinghai-Tibetan Plateau Animal Genetic Resource Reservation and Utilization Key Laboratory of Sichuan Province, Southwest Minzu University, Chengdu 610000, China; 3 Mammalian NutriPhysioGenomics, Department of Animal Sciences and Division of Nutritional Sciences, University of Illinois, Urbana, IL 61801, USA; qj5@illinois.edu

**Keywords:** gene editing, lactation, lipid metabolism, milk fat, ruminant

## Abstract

In nonruminants, microRNA (miRNA)-24 plays an important role in lipid metabolism in adipose tissue and the liver. Although the abundance of miR-24 in ruminant mammary glands is the highest during peak lactation, its potential role in regulating the synthesis and secretion of fat into milk is unclear. This study aimed to identify the function of miR-24 in these processes using CRISPR/Cas9 technology in primary goat mammary epithelial cells (GMEC). A single clone containing a 66-nucleotide deletion between two sgRNAs mediating double-strand break (DSB) sites was obtained. The abundance of miR-24-3p and miR-24-5p encoded by the deleted sequence was decreased, whereas the target genes *INSIG1* and *FASN* increased. In addition, miR-24 knockout reduced the gene abundance of genes associated with fatty acid and TAG synthesis and transcription regulator. Similarly, the content of cholesterol and monounsaturated fatty acid (MUFA) C18:1 decreased, whereas that of polyunsaturated fatty acids (PUFA) C18:2, C20:3, C20:4 and C20:5 increased. Subsequently, knocking down of *INSIG1* but not *FASN* reversed the effect of miR-24 knockout, indicating that miR-24 modulated cholesterol and fatty acid synthesis mainly by targeting *INSIG1*. Overall, the present in vitro data demonstrated a critical role for miR-24 in regulating lipid and fatty acid synthesis and highlighted the possibility of manipulating milk components in dairy goats.

## 1. Introduction

Goat milk has been considered a nutraceutical for decades because of its smaller fat globule, higher fat content and lower allergenic properties than cow milk [1]. Ninety-eight percent of milk fat is TAG, and the remainder is composed of cholesterol and free fatty acids [2]. Compared with cow milk, short- and medium-chain fatty acids (FA) and unsaturated FA contents in the TAG of goat milk are higher [3]. Monounsaturated fatty acid (MUFA) provides energy to the young and has anti-cardiovascular and anti-inflammatory effects in adults [4]. Similarly, cholesterol is responsible for growth and development throughout life, and its homeostasis is vital in numerous diseases [5,6].

Previous studies have shown that microRNA (miRNA) abundance is associated with fatty acid composition in goat milk and can regulate lipid synthesis in primary GMEC [7,8]. Small-RNA sequencing data have revealed that the expression of miR-24 is upregulated in the differentiation process in bovine preadipocytes, and this, combined with the following target gene research, shows that miR-24 plays a crucial role in lipogenesis [9]. In non-ruminants, the abundance of microRNA-24 is high in human hepatocytes, and its knockdown in mice impairs lipid synthesis in the liver when they are fed a high-fat diet for 12 weeks [10]. In non-ruminants, at least one target partly responsible for the effects on lipid metabolism induced by miR-24 is *FABP4* [11]. Thus, the available data underscore the critical role of miR-24 in regulating various aspects of lipid metabolism.

Fatty acid synthase protein regulates the synthesis of short, medium-chain, and C16 FAs [12]. Polymorphisms of *FASN* contribute to the variation in milk fat content in Holstein cows, indicating that this gene is a candidate in the QTL region for milk fat [13]. Epigenetic downregulation of *FASN* inhibits de novo lipogenesis in visceral adipose tissue from obese subjects, thereby leading to the development of T2D [14]. In addition, reducing *FASN* expression inhibits C10:0 and C12:0 fatty acids synthesis in GMEC [15]. The role of FASN in fatty acid synthesis is unquestionable.

The insulin-induced gene 1 (INSIG1) protein negatively regulates mammary lipid synthesis b, inhibiting the cytoplasmic processing of SREBP-1 protein [16]. A previous study in transgenic mice indicated that plasma cholesterol and TAG were reduced upon refeeding on a high-carbohydrate diet when there was liver-specific overexpression of human INSIG1 [17]. In ruminants, overexpression of *INSIG1* reduces the expression of fatty acid metabolism-related genes, thereby reducing TAG content in buffalo mammary cells [18].

Its ease of use has made CRISPR/Cas9 the most commonly used system for genetic editing in a wide variety of species [19]. sgRNA allows targeting of Cas9 to specific loci, and the endogenous miR-locus is destroyed and/or excised with a dual sgRNA, resulting in a stable miRNA knockout (KO) phenotype in vitro and in vivo [20]. Combined with transgenic technology, this could efficiently promote the distribution of desirable traits of miRNA editing in animal populations. The use of in vitro transfected nuclear donor cells, whose miRNA transcriptomes have been successfully edited using the CRISPR/Cas9 strategy, for the purposes of somatic cell nuclear transfer (SCNT)-mediated assisted reproductive technologies appears to be a very promising approach aimed at efficiently propagating and multiplying genetically identical specimens in dairy breeds of livestock species characterized by highly heritable and functionally relevant quantitative traits that determine the biochemical composition and quality-related parameters of milk [21,22,23,24,25,26]. Thus, the knockout of miRNA using CRISPR/Cas9 technology can more accurately elucidate the effect of a given miRNA and its coding sequence.

These findings led us to hypothesize that miR-24 alters lipid and fatty acid synthesis by targeting *INSIG1* in GMEC. To test this hypothesis, stable miR-24 knockout GMEC were generated using CRISPR/Cas9 technology. This study aimed to elucidate the mechanisms by which miR-24 regulates lipid and fatty acid synthesis.

## 2. Materials and Methods

### 2.1. Animal and Cell Culture

All animal experimental procedures were approved by the Institutional Animal Care and Use Committee of the University of Northwest A&F, Yangling, Shaanxi, China following the guidelines for experimental animals (permit number: 15-516, date: 13 September 2015).

The GMEC were isolated from mammary gland biopsies of five three-year-old healthy Xinong Saanen dairy goats during peak lactation (120 d post parturition). Briefly, goats were anesthetized by intravenous injection of xylazine hydrochloride (0.01 mL/kg, Huamu Animal Health Products Corporation, Shandong, China) before surgery. Mammary tissues were obtained from each goat and washed with diethylpyrocarbonate (DEPC)-treated phosphate-buffered saline (PBS) until the blood was removed. Tissues were obtained under sterile conditions. Subsequently, the connective and fat tissues were trimmed, and the parenchyma was rinsed twice with D-Hank’s solution. The mammary alveoli isolated from the samples were then minced and mixed into approximately 1 mm^3^ tissue blocks and plated in a pretreated 60 mm culture dish to separate the GMEC. Cells were cultured individually and purified for five passages according to the different adhesion properties and frozen according to published procedures in our laboratory [27]. The purified GMEC were identified by microscopy, immunofluorescence analysis of cytokeratin 18 and vimentin, and Western blot analysis of CSN2 (Supplementary Figure S1). The GMEC from three of five goats were used in subsequent experiments.

The GMEC from three goats were cultured individually in a basal medium in an environment of 5% CO_2_ at 37 °C, as described previously by our group [28]. Basal medium consisted of 90% DMEM/F12 (SH30023−01, Hyclone, Logan, UT, USA) and 10% fetal bovine serum (10099-141, Invitrogen) supplemented with 5 μg/mL bovine insulin (16634, Sigma, St. Louis, MO, USA), 100 U/mL penicillin/streptomycin (080092569, Harbin Pharmaceutical Group, Harbin, China), 5 μg/mL hydrocortisone (H0888, Sigma, St. Louis, MO, USA) and 10 ng/mL epidermal growth factor (PHG0311, Invitrogen, Waltham, MA, USA). To induce lactation, the cells in the dish were treated with 2 μg/mL prolactin (L6520, Sigma, St. Louis, MO, USA) prior to functional investigation for 48 h.

### 2.2. Immunofluorescence Analysis

The GMEC obtained from the three goats were individually seeded in plates, followed by washing three times with PBS buffer. Then, cells in the plates were fixed in 4% paraformaldehyde (PFA) and made permeable at 4 °C using 0.5% Triton-X100 in PBS buffer. Subsequently, the cells were blocked with 5% BSA (180728, MP Biomedicals, Santa Ana, CA, USA) in PBS for 1 h followed by incubation with primary antibodies against cytokeratin 18 (66187-1-Ig, Proteintech Group, Wuhan, China; 1:100) and vimentin (ab8069, Abcam, Cambridge, MA, USA, 1:500) for 12 h at 4 °C. Alexa Fluor 488-conjugated AffiniPure goat anti-rabbit IgG and goat anti-mouse IgG (SA00006−2, SA00013−1, Proteintech Group; 1:200) were incubated with secondary antibodies in 5% BSA for 1.5 h. Nuclei were stained with 2 μg/mL DAPI in PBS for 5 min. Each step was followed by washing three times with PBS buffer. Images were acquired using a cell imaging detector (BioTek Instruments Inc., Palo Alto, CA, USA).

### 2.3. Construction of Cas9-sgRNAs Plasmid

sgRNAs targeting the goat pre-miR-24 genomic sequence were designed using the Guide RNA web tool (http://chopchop.cbu.uib.no/ (accessed on 11 November 2020)). Two sgRNAs targeting both ends of the pre-miR-24 genomic sequence were selected for the Cas9-sgRNA1-sgRNA2 vector construction. The plasmid pSpCas9 (BB)-2A-Puro (62988, Addgene, Cambridge, MA, USA) was chosen as the backbone and used as a control. The double-stranded sequences of sgRNA1 and sgRNA2 were annealed by two complementary single-strand oligonucleotides from Invitrogen corporation (Shanghai, China). Thereafter, the double-stranded fragments of sgRNA1 and sgRNA2 were inserted at the *Bbs*I site into the plasmid pX459 to construct pX459-sgRNA1 and pX459-sgRNA2 plasmids. The U6-sgRNA2-tracRNA sequences from pX459-sgRNA2 were amplified by PCR using the following primers: forward 5′-CACCTCTAGAGAGGGCCTATTTCCCATGATTCCTTCATAT-3′ and reverse 5′-CACCGGTACAAAAAAGCACCGACTCGGTGCCACTTTTTC-3′. The PCR fragment was then cloned at *Xba*I-*Kpn*I sites into the plasmid pX459-sgRNA1 to generate the Cas9-sgRNA1-sgRNA2 plasmid, which was used to select single-cell clones and subsequent functional verification.

### 2.4. Cell Transfection, Single Clone Selection and Validation

Primary mammary cells were transfected with Cas9-sgRNA1-sgRNA2 or pX459 vectors at 70–80% confluence using Lipofectamine 2000 (11668019, Invitrogen), following the manufacturer’s instructions. After transfecting for forty-eight hours, the GMEC were treated with puromycin (puro) (P8833, Sigma) for 96 h at 1.0 μg/mL concentrations. The surviving cells were then diluted and expanded for single-clone selection. When the cell density reached 90–100% confluence in a 48-well plate, 50 percent of the cells were used for genome extracting. The DNA fragments spanning the pre-miR-24 sequence were amplified by PCR reaction using the following primers: forward 5′-AGTCAAGCAAGACTATGTCAGCGGTAGATG-3′ and reverse 5′-GCCTGTTTCAGACCCACAGTCATTCC-3΄. The amplicon was purified and submitted to T7EN1 and Sanger sequencing. Detailed procedures for single-cell selection and the cleavage frequency analysis have already been described in previous publications [29]. After selecting three single clones with the same nucleotide mutation, we conducted subsequent experiments in these three single clones.

To inhibit *FASN* expression, three small interfering RNAs (siRNAs) (siFASN-369, siFASN-4783, and siFASN-5395) from Gene Pharma (Shanghai, China) were designed to obtain an ideal knockdown efficiency (Supplementary Figure S2A). miR-24 knockout and control cells were transfected using Lipofectamine^TM^ RNAiMAX reagent (13778075, Invitrogen) with 50 n*M* siFASN, siNC or siINSIG1, following the manufacturer’s specifications. The siNC and siINSIG1 were also ordered from Gene Pharma, and the RNA sequences of all siRNAs are displayed in Supplementary Table S1. The siINSIG1 sequence was synthesized as previously reported [30].

### 2.5. Off-Target Effect Analysis in Knockout Cells

Off-target (OT) sites guided by sgRNA1 and sgRNA2 were analyzed using CRISPR RGEN Tools Cas-OFFinder. Thereafter, the off-target sites with mismatches ≤ 4 bp that were induced by sgRNA1 and sgRNA2 were selected. Genomes from control and knockout cells were extracted, and PCR products of genomic sequences were used for off-target effect analysis via the T7EN1-cleavage assay and Sanger sequencing. Primer sequences for amplifying the sequences containing the OT sites are reported in Supplementary Table S2.

### 2.6. Reverse Transcription Quantitative PCR (RT-qPCR)

Cellular RNA from GMEC was extracted using the TRIzol reagent (9109, Takara) following the manufacturer’s instructions. RNA quality was examined by concentration detection, in which the ratio of OD260/OD280 was 1.8–2.0, and OD260/OD230 was 2.0–2.2. Integrity was determined through analysis of 18S and 28S rRNA analysis by gel electrophoresis (data not shown). miRNA expression was determined using 0.5 μg total RNA by CFX-96 Touch Real-Time PCR Detection System (Bio-Rad Laboratories Inc., Hercules, CA, USA) using the S-Poly (T) Plus method following the manufacturer’s specification [31]. The relative abundance of miRNAs was calculated and normalized to that of 18S rRNA using the 2^−ΔΔCt^ method. All the miRNA primers used are listed in Supplementary Table S3.

mRNA abundance was examined using the PrimeScript RT Reagent Kit (RR820A, Perfect Real Time, Takara) from 0.5 μg total RNA. Genomic DNA was removed using the gDNA eraser enzyme. After reverse transcription, the cDNA was subjected to qPCR detection using SYBR Premix Ex TaqII reagent. Relative mRNA abundance was calculated by normalizing to the ribosomal protein S9 (*RPS9*) and ubiquitously expressed transcript (*UXT*) using the 2^−ΔΔCt^ method. All the primers used for mRNA quantification are listed in Supplementary Table S4.

### 2.7. Protein Extraction and Western Blot

To extract total protein from GMEC, cells were washed and lysed with RIPA buffer (R0010, Solarbio Tech Inc., Beijing, China) supplemented with protease inhibitor cocktail tablets (04693132001, Roche., Mannheim, Germany). Detailed procedures of Western blotting are reported in one of our previous studies [32]. The primary antibodies used were rabbit anti-β-casein (bs-0466R, Bioss, Woburn, CA; 1:1000), rabbit anti-INSIG1 (bs-5074R, Bioss; 1:500), rabbit anti-FASN (10624-2-AP, Proteintech Group, Wuhan, China; 1:500), and mouse anti-β-tubulin (CW0098, CW Biotech, Beijing, China; 1:1000). Secondary antibodies included horseradish peroxidase (HRP)-conjugated goat anti-mouse_IgG (CW0102, CW Biotech; 1:4000) and HRP-conjugated goat anti-rabbit_IgG (CW0103, CW Biotech; 1:4000). Protein abundance was measured using the ImageJ software. The relative abundance of FASN and INSIG1 proteins was calculated by normalizing to *β*-Tubulin.

### 2.8. BODIPY™ 493/503 Staining

The GMEC in the plates were washed and fixed in 4% PFA solution for 0.5 h. Lipid droplets in GMEC were stained using 0.1% BODIPY 493/503 reagent (Invitrogen) in DMSO for 30 min. The nuclei were counterstained with DAPI. Staining steps were followed by washing three times with PBS. The GMEC images were acquired using a cell imaging detector (BioTek Instruments Inc., Palo Alto, CA, USA). The relative BODIPY values were normalized by comparison with DAPI.

### 2.9. Triacylglycerol and Cholesterol Assays

TAG and cholesterol contents in GMEC were quantified using a triacylglycerol enzymatic assay kit (E1013, Applypen Technologies Inc., Beijing, China) and cholesterol enzymatic assay kit (E1015, Applygen Technologies Inc., Beijing, China) following the manufacturer’s instructions. Cellular TAG and cholesterol content is shown as the ratio of TAG or cholesterol concentration to the protein concentration. Detailed procedures for these assays have been reported previously [33]. 

### 2.10. Fatty Acid Analysis

The GMEC were seeded in dishes, and total fatty acids were extracted with 2 mL of 2.5% (*v*/*v*) sulfuric acid/methanol. Fatty acid extraction and analysis were performed according to protocols in our laboratory [15]. The proportion of each fatty acid is shown as the ratio of the total fatty acids that could be detected. Prior to determining the fatty acid composition, we used the Supelco 37 Component FAME Mix reagent (CRM47885, Sigma St. Louis, MO, USA) for peak identification.

### 2.11. Statistical Analysis

Data are shown as the means ± SEM. All independent experiments were conducted using individual GMEC from the three goats. Independent experiments were repeated thrice on consecutive days. Statistical analysis was performed using Student’s *t*-test for double comparisons or one-way ANOVA for multiple comparisons with SPSS 19.0 (SPSS, Inc., Chicago, IL, USA). *p* < 0.05 was determined as statistically significant.

## 3. Results

### 3.1. Single-Cell Clone Selection and Editing Efficiency

**Figure 1:**
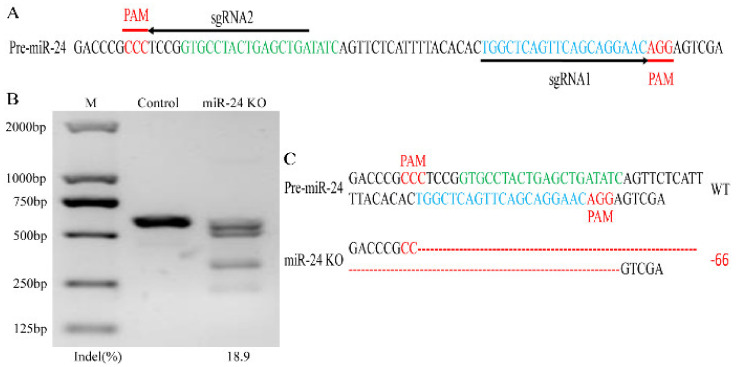
sgRNA design of the genomic sequence of pre-miR-24 in Capra hircus and gene editing analysis of single clone. (**A**) The corresponding genomic sequence of pre-miR-24 and sgRNAs with its targeted sites. The sgRNA1 and sgRNA2 are shown by horizontal arrowheads (black). The PAM sequence of sgRNAs is displayed in underline and red, and the corresponding DNA sequence of miR-24-3p and 5p is shown in green and blue. (**B**) The efficiency of T7EN1 cleavage reaction towards pre-miR-24 genome in the single clone and quantified analysis by bands density using ImageJ. (**C**) Sequence of pre-miR-24 genome in the single-cell clone. Wild type (WT) and deletion (-) are presented on the right.

Through sgRNAs prediction of the pre-miR-24 genome, sgRNA1 and sgRNA2, which were predicted to target the miR-24-3p and miR-24-5p genomic sequences, were selected (Figure 1A). After transfection of two sgRNA co-expressing vectors and following single-clone selection, we obtained a single clone with 66 nucleotide deletion between the two double-strand break (DSB) sites (Figure 1C). As shown in Figure 1B, the single-cell clone showed 18.9% cleavage efficiency around the pre-miR-24 genomic sequence (Figure 1B).

### 3.2. Off-Target Effect Analysis Mediated by sgRNAs

**Figure 2:**
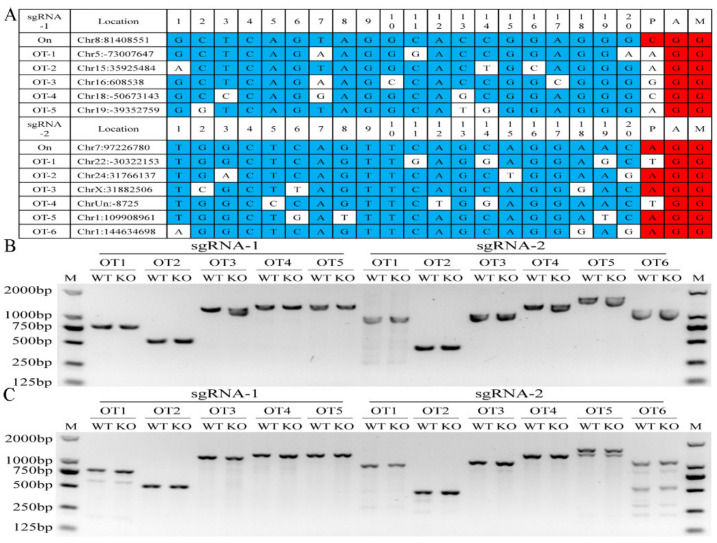
Detection of Cas9 protein mediating off-target effect caused by sgRNA1 and sgRNA2 in GMECs. (**A**) On-target and corresponding off-target sequences along with their PAM motif and genomic location in Capra hircus genome. The nucleotides of sgRNA and PAM sequences are displayed in red and blue, respectively. (**B**) Determination of the PCR products spanning the sequence of off-target. OT, off-target; WT, wild type site. (**C**) Detection of cleavage efficiency of off-target sites by T7EN1-cleavage assay.

Among the predicted off-target sites in the goat genome mediated by sgRNA1 and sgRNA2, there were five sgRNA1 and six sgRNA2 OT sites, which were selected for verification (Figure 2A). PCR results showed that the bands for off-target sites were specific to miR-24 KO and control cells (Figure 2B). The amplified products were subjected to T7EN1 cleavage assay. Figure 2C shows that only the OT6 site of sgRNA2 produced cleavage bands. The sequence of the amplified product was validated by Sanger sequencing, and the results in Supplementary Figure S3 show that two single nucleotide mutations and one single nucleotide deletion were present in the genomes of miR-24 KO and control cells.

### 3.3. Deletion of miR-24 Sequence Decreases miR-24-3p and 5p Abundance and Increases FASN and INSIG1 Abundance

**Figure 3:**
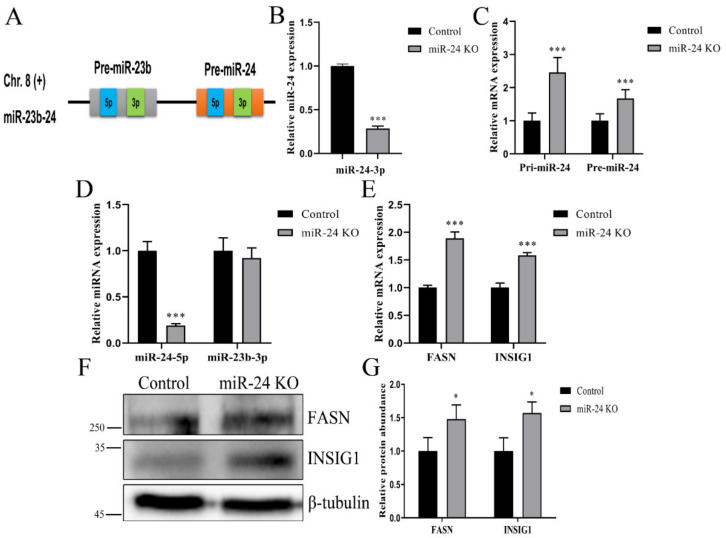
Knockout of miR-24 decreases miRNA abundance and increases *INSIG1* and *FASN* abundance. (**A**) Abundance analysis of miR-24-3p by RT-qPCR in control and knockout cells. (**B**) Detecting the mRNA abundance of pri- and pre-miR-24 sequence in knockout cells. (**C**) Abundance analysis of miR-24-5p and miR-23b-3p in knockout cells. (**D**) mRNA abundance analysis of *INSIG1* and *FASN* in control and knockout cells. (**E**,**F**) Abundance analysis of INSIG1 and FASN proteins in control and miR-24 knockout cells by Western blot. Data are shown as mean ± SEM in these repeated experiments. * *p* < 0.05, *** *p* < 0.001.

To elucidate the effect of the 66-nucleotide deletion of the pre-miR-24 genomic sequence on the abundance of miR-24-3p, RT-qPCR assay was conducted and the miR-24-3p abundance in the miR-24 knockout group decreased to 28.6% compared with control cells (*p* < 0.01, Figure 3A). To ascertain the effect of nucleotide deletion on miRNA biosynthesis, we examined the abundance of pri- and pre-miRNAs and found an increase in both pri-miR-24 and pre-miR-24 levels (*p* < 0.01, Figure 3B). Subsequently, we detected miR-24-5p abundance and found that miR-24 knockout decreased the miR-24-5p abundance (*p* < 0.01, Figure 3C). The miR-23b genomic sequence is clustered with miR-24, and the miR-23b-3p abundance was not altered after deletion of the miR-24 coding sequence (*p* > 0.05, Figure 3C).

In this study, miR-24 knockout promoted the mRNA expression of *INSIG1* and *FASN* (*p* < 0.01, Figure 3D). As expected, the protein abundance of INSIG1 and FASN were both increased upon knocking out miR-24 (*p* < 0.01, Figure 3E,F).

### 3.4. Knockout of miR-24 Inhibits Lipid Droplets, TAG and Cholesterol Synthesis and Affects Fatty Acid Composition

**Figure 4:**
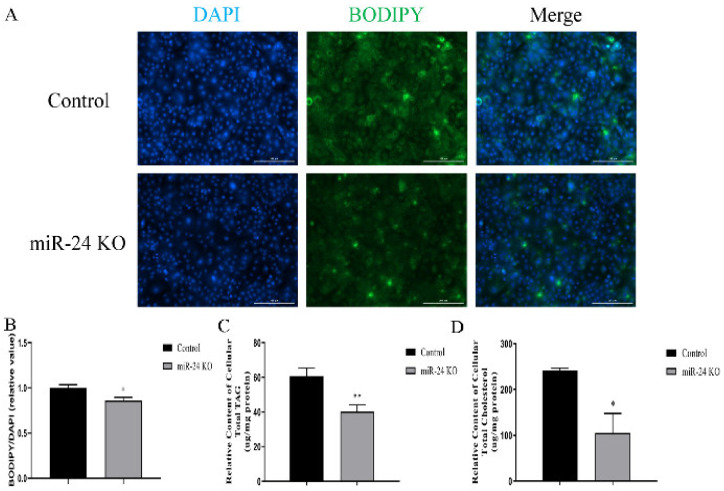
miR-24 knockout inhibits lipid droplet, TAG and cholesterol accumulation. The contents of lipid droplet (**A**,**B**), TAG (**C**) and cholesterol (**D**) in control and miR-24 knockout cells. Data are expressed as mean ± SEM in these repeated experiments. * *p* < 0.05, ** *p* < 0.01.

Cellular lipid droplet content decreased upon miR-24 knockout (*p* < 0.05, Figure 4A,B). Moreover, relative TAG and cholesterol concentrations were both decreased in the knockout group (*p* < 0.05, Figure 4C,D). After detecting the fatty acid profile in miR-24 knockout cells, the percentages of C17:0, C18:0 and C18:2 (*p* < 0.05) increased, whereas C18:1 (*p* < 0.01) decreased. Among the content of PUFA in GMEC, the percentages of C20:3, C20:4, and C20:5 increased. On this basis, the proportions of saturated fatty acids (SFA) and PUFA were both increased, whereas MUFA decreased in knockout cells (Table 1).

### 3.5. Knocking Out miR-24 Reduces Fatty Acid Metabolism-Related Gene Expression

**Figure 5:**
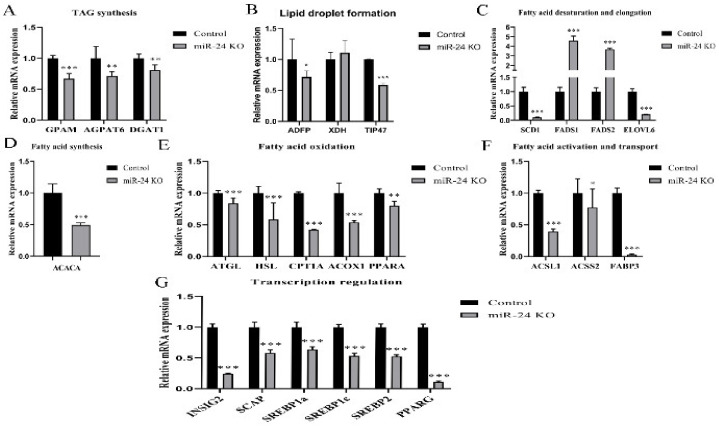
Knockout of miR-24 decreases the abundance of genes associated with fatty acid metabolism. mRNA abundance of genes of TAG synthesis (**A**), lipid droplet formation (**B**), fatty acid desaturation and elongation (**C**), fatty acid synthesis (**D**), fatty acid oxidation (**E**), fatty acid activation and transport (**F**) and transcription regulation (**G**) in control and miR-24 knockout cells. Data are presented as mean ± SEM in these repeated experiments. * *p* < 0.05, ** *p* < 0.01, *** *p* < 0.001.

Knockout of miR-24 decreased the abundance of TAG synthesis-related genes *GPAM*, *AGPAT6* and *DGAT1* and lipid droplet formation-related genes *ADFP* and *TIP47* (*p* < 0.05). The abundance of the fatty acid desaturation and elongation genes *SCD1* and *ELOVL6* along with the fatty acid synthesis gene *ACACA* decreased after miR-24 knockout (*p* < 0.001). The abundance of lipolysis- and fatty acid oxidation-related genes *ATGL*, *HSL*, *CPT1A*, *ACOX1* and *PPARA* was downregulated by the miR-24 knockout (*p* < 0.01). The abundance of fatty acid activation and transport genes *ACSL1*, *ACSS2* and *FABP3* was downregulated in knockout cells (*p* < 0.05). In addition, the transcription of transcription factors and co-factors *INSIG2*, *SCAP*, *SREBP1a*, *SREBP1c*, *SREBP2* and *PPARG* was decreased in knockout cells (*p* < 0.001). In contrast, miR-24 knockout increased the abundance of very long-chain fatty acid desaturation genes *FADS1* and *FADS2* (*p* < 0.001, Figure 5).

### 3.6. Knocking Out miR-24 Inhibits Lipid Droplets, TAG and Cholesterol Synthesis and Affects Fatty Acid Content Mainly via Targeting INSIG1

**Figure 6:**
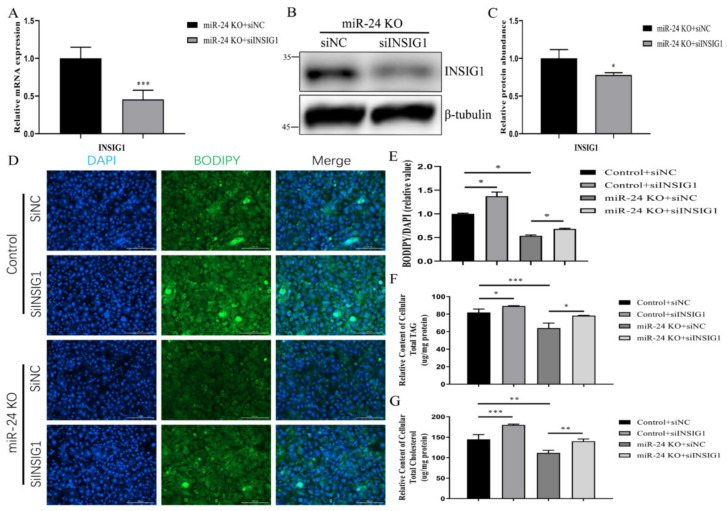
Knockout of miR-24 inhibits lipid droplet, TAG and cholesterol accumulation via targeting *INSIG1*. (**A**) Abundance analysis of *INSIG1* mRNA in miR-24 knockout cells transfected with siINSIG1 or siNC. (**B**,**C**) INSIG1 protein abundance analysis in miR-24 knockout cells transfected with siINSIG1 or siNC by Western blot. (**D**–**G**) The contents of lipid droplet (**D**,**E**), TAG (**F**) and cholesterol (**G**) in control and miR-24 knockout cells transfected with siINSIG1 or siNC. Data are expressed as mean ± SEM in these repeated experiments. * *p* < 0.05, ** *p* < 0.01, *** *p* < 0.001.

Transfection with siFASN reduced the protein abundance of FASN (*p* < 0.05, Supplementary Figure S2B,C). Silencing of *FASN* resulted in a decreased TAG content (*p* < 0.05, Supplementary Figure S2D). As shown in Figure 6A–C, transfection with siINSIG1 decreased the abundance of *INSIG1* mRNA and protein (*p* < 0.05). Furthermore, a lower abundance of *INSIG1* reversed the miR-24 knockout-induced downregulation of droplet content (*p* < 0.05, Figure 6D,E). In addition, the decreased TAG and cholesterol contents caused by miR-24 knockout were rescued by *INSIG1* expression interference in knockout cells (*p* < 0.05, Figure 6F,G).

Among the total fatty acids detected in the cells, the percentages of C16:0 and C16:1 were restored after *INSIG1* silencing in miR-24 knockout cells (*p* < 0.05). The increase in the percentage of C18:0 was reversed by *INSIG1* expression interference (*p* < 0.05). The decreased percentage of C18:1 and the increased percentage of C18:2 further decreased and increased, respectively, as a consequence of *INSIG1* silencing (*p* < 0.05). Among the C20 PUFA detected in GMEC, only C20:5 showed an increase in percentage upon inhibition of *INSIG1* abundance (*p* < 0.05). After knocking down *INSIG1* in knockout cells, the ratio of MUFA and PUFA was further decreased and increased, respectively (Table 2).

## 4. Discussion

In our previous study, deleting one nucleotide adjacent to the Drosha cleavage site with a single sgRNA inhibited mature miR-145 abundance by suppressing pri-miRNA processing [32]. However, in this study, we knocked out miR-24 via two sgRNAs targeting both strands and miR-24-3p and 5p sequences of the pre-miR-24 genome. A single clone with a 66-nucleotide deletion between the target sites of the two sgRNAs was obtained. T7EN1 and subsequent Sanger sequencing assays revealed no evidence of gene modification in the off-target sites of the two sgRNAs, which was similar to a previous observation [34].

The double-guide RNAs-induced CRISPR/Cas9 system successfully produced an miR-24 knockout single clone with a large fragment deletion, which was consistent with the miR-160a knockout detected in Arabidopsis [35]. Pre-miRNA-specific sequences and the stem-loop structure folded by the nascent transcript affect miRNA biogenesis [36]. The increase in the abundance of pri-miR-24 and pre-miR-24 when miR-24 was knocked out confirmed the importance of the pre-miR-24 sequences. The pri-miRNAs and pre-miRNAs are processed by Drosha and Dicer endonucleases and their partner proteins to generate the miRNA duplex. Thus, disruption of miRNA biogenesis affects the expression of 5p and 3p strands of miRNAs [37]. Subsequently, we detected the abundance of miR-24-5p, complemented to miR-24-3p, and found the abundance decreased, which supports the finding in pri- and pre-miRNA expressions. As miRNAs are often transcribed in clusters and interdependency in abundance does not seem to occur in all clustered miRNAs [38,39], we detected the abundance of miR-23b, indicating that the 66-nucleotide deletion in the pre-miR-24 genome did not affect its transcription.

Intracellular fatty acids comprise a large portion of the TAG stored in the cytoplasm, and TAG, along with cholesterol, is the main lipid species in mammary cells [40]. The inhibition of lipid droplets, TAG and cholesterol in miR-24 knockout cells was in agreement with the effect of miR-24 knockdown using chemical inhibitors [41]. It is well known that GPAM, AGPAT6 and DGAT1 play distinct functional roles in TAG biosynthesis [42]. This study showed that the downregulation of these three genes was in line with the lower concentration of TAG in miR-24 knockout cells. SREBP2 and SREPB1a work in concert to regulate the abundance of genes associated with cholesterol formation [43]. Accordingly, the intracellular concentration of cholesterol may be related to the abundance of these proteins. Lipid droplets contain a neutral lipid core, which is mainly composed of TAG [44] and the proteins ADFP, XDH, and TIP47, which are involved in the formation and secretion of the lipid droplets [45]. Thus, the decrease in concentrations of TAG and cholesterol, combined with the downregulation of *ADFP* and *TIP47* in miR-24 knockout cells, partly explained the lower lipid droplet content.

Most short- and medium-chain FA and palmitate acid (C16:0) are produced via synthesis regulated by ACACA and FASN [33]. The C16:0 to C18:0 fatty acid conversion is catalyzed by ELOVL6 [46]. The increase in the percentage of C18:0 in the knockout cells may have been caused by the effects observed on ACACA, FASN, and ELVOL6. *SCD1* encodes a desaturase that catalyzes the conversion of C18:0 to C18:1 [47]. In contrast, C18:2 is produced via desaturation catalyzed by FADS2 [48]. The decrease in C18:1 percentage and the increase in C18:2 appeared to be a consequence of the downregulation of *SCD1* and upregulation of *FADS2*. As very-long-chain PUFA are synthesized via desaturation of very-long-chain fatty acids catalyzed by FADS1 and FADS2, the upregulation of *FADS1* and *FADS2* could lead to an increase in the percentage of C20:3, C20:4, and C20:5 in knockout cells.

Both *INSIG1* and *FASN* are target genes of miR-24, which can control fatty acid and TAG synthesis in mice and goats [10,41]. Thus, a more comprehensive survey is warranted to elucidate the roles of *FASN* and *INSIG1* in miR-24 regulating lipid and fatty acid synthesis. The decrease in TAG content after knockdown of *FASN* expression via siRNA transfection in miR-24 knockout cells was in accordance with previous findings in GMEC [33]. These results demonstrate that miR-24 targets *FASN* and promotes lipid and fatty acid synthesis but knocking out miR-24 reduces TAG and cholesterol content, indicating that miR-24 regulation of lipid and fatty acid synthesis is also affected by other genes. For example, after knockdown of *INSIG1*, the negative effect on fatty acid synthesis caused by miR-24 knockout was reversed. Thus, *INSIG1* inhibition via miR-24 likely plays a critical role in fatty acid synthesis. Because *INSIG1* can inhibit the abundance of *SREBP1* and suppress target genes associated with fatty acids and TAG synthesis [49], we speculate that miR-24 regulates lipid and fatty acid synthesis mainly by targeting *INSIG1*.

## 5. Conclusions

In vitro, the abundance of miR-24 in goat mammary cells can control lipid droplet, TAG, and cholesterol content, along with fatty acid composition, particularly medium- and long-chain fatty acids. These effects were associated with the abundance of *INSIG1* and *FASN*. Thus, our study provides evidence for an important biological role of miR-24 in various aspects of lipid metabolism in goat mammary cells. Further studies are necessary to clarify the underlying molecular mechanism and help form a theoretical frame of reference for modifying the beneficial fatty acid content of goat milk. The use of genomic nucleotide deletion through sgRNA, along with other molecular tools, could aid these efforts.

## Figures and Tables

**Table 1:** Fatty acid composition by GC-MS in Control and miR-24 KO GMECs.

Fatty Acid (%)	Control	miR-24 KO
C14:0	2.65 ± 0.32	2.90 ± 1.01
C15:0	0.29 ± 0.04	0.35 ± 0.03
C16:0	51.63 ± 1.58	51.38 ± 1.03
C16:1	1.16 ± 0.18	1.48 ± 0.14
C17:0	0.61 ± 0.11 ^b^	0.85 ± 0.03 ^a^
C17:1	0.32 ± 0.06	0.32 ± 0.03
C18:0	23.59 ± 0.6 ^b^	25.39 ± 0.88 ^a^
C18:1	16.91 ± 0.45 ^A^	13.54 ± 0.79 ^B^
C18:2	0.74 ± 0.12 ^b^	1.06 ± 0.05 ^a^
C20:0	0.27 ± 0.03	0.32 ± 0.04
C20:3	0.34 ± 0.03 ^b^	0.43 ± 0.02 ^a^
C20:4	0.96 ± 0.07 ^b^	1.24 ± 0.09 ^a^
C20:5	0.18 ± 0.01 ^B^	0.41 ± 0.03 ^A^
C22:6	0.37 ± 0.02	0.33 ± 0.03
SFA	79.03	81.18
MUFA	18.39	15.35
PUFA	2.58	3.47

Fatty acid data are showed as proportions of total fatty acids. Statistical significance between wild-type (Control) and miR-24 knockout (KO) is as follows: *p* < 0.05 expressed as lowercase letters and *p* < 0.01 as uppercase letters, and the a/A letters were located in the large value in each kind of fatty acids between the two group. Data are shown as mean ± SEM.

**Table 2:** Effects of INSIG1 knockdown on miR-24 KO-mediating changes in fatty acid composition.

Fatty Acid (%)	Control + siNC	Control + siINSIG1	miR-24 KO + siNC	miR-24 KO + siINSIG1
C14:0	4.25 ± 0.32 ^a^	4.07 ± 0.12 ^a^	2.70 ± 0.14 ^b^	2.67 ± 0.31 ^b^
C15:0	0.50 ± 0.05 ^b^	0.52 ± 0.01 ^ab^	0.51 ± 0.01 ^ab^	0.58 ± 0.04 ^a^
C16:0	51.04 ± 0.48 ^a^	49.61 ± 0.08 ^b^	45.62 ± 0.32 ^d^	46.94 ± 0.66 ^c^
C16:1	1.60 ± 0.05 ^b^	1.61 ± 0.01 ^b^	1.56 ± 0.05 ^b^	1.70 ± 0.02 ^a^
C17:0	0.85 ± 0.05 ^c^	0.93 ± 0.04 ^b^	1.07 ± 0.02 ^a^	1.08 ± 0.03 ^a^
C17:1	0.43 ± 0.05 ^ab^	0.46 ± 0.03 ^a^	0.33 ± 0.00 ^c^	0.37 ± 0.03 ^bc^
C18:0	21.22 ± 0.24 ^d^	21.93 ± 0.11 ^c^	26.84 ± 0.14 ^a^	24.96 ± 0.32 ^b^
C18:1	15.69 ± 0.17 ^a^	15.58 ± 0.37 ^a^	14.35 ± 0.17 ^b^	13.97 ± 0.43 ^b^
C18:2	1.32 ± 0.09 ^d^	1.50 ± 0.04 ^c^	2.76 ± 0.15 ^b^	3.01 ± 0.03 ^a^
C20:0	0.36 ± 0.02 ^c^	0.42 ± 0.02 ^b^	0.51 ± 0.06 ^a^	0.56 ± 0.04 ^a^
C20:3	0.52 ± 0.02 ^c^	0.64 ± 0.01 ^b^	0.71 ± 0.08 ^ab^	0.78 ± 0.04 ^a^
C20:4	1.51 ± 0.14 ^b^	1.83 ± 0.10 ^a^	1.91 ± 0.16 ^a^	2.12 ± 0.18 ^a^
C20:5	0.29 ± 0.01 ^d^	0.36 ± 0.01 ^c^	0.75 ± 0.07 ^b^	0.86 ± 0.03 ^a^
C22:6	0.42 ± 0.03 ^b^	0.54 ± 0.03 ^a^	0.37 ± 0.04 ^b^	0.41 ± 0.03 ^b^
SFA	78.23	77.48	77.26	76.78
MUFA	17.71	17.66	16.24	16.03
PUFA	4.06	4.87	6.50	7.18

Statistical analysis among these four groups was assessed by one-way ANOVA with Duncan’s test. Statistical significance is as follows: lower case letters, *p* < 0.05, and the different lower case letters represent significant difference between any two groups. Data are shown as mean ± SEM.

## Data Availability

Not applicable.

## Supplementary Materials

The following supporting information can be downloaded at: https://www.mdpi.com/article/10.3390/foods11142012/s1, Figure S1: Identification of primary goat mammary epithelial cells by immunofluorescence staining and western blot [41]; Figure S2: Interference with FASN inhibits TAG synthesis in miR-24 knockout cells [50]; Figure S3: Sequences of two alleles in off-target site 6 of sgRNA2 [51]; Table S1: The siRNA Sequences of *FASN* and *INSIG1* [15]; Table S2: Primers Used for 11 Off-Target Sites Detection of sgRNAs [43]; Table S3: Primers for Reverse Transcription Quantitative PCR of miRNAs [52,53]; Table S4: Characteristics of Primers for mRNA RT-qPCR [29,30].

## Abbreviations

GMECs	goat mammary epithelial cells
miRNA	microRNA
CRISPR/Cas9	clustered regularly interspaced short palindromic repeats/CRISPR associated nuclease 9
DSB	double-strand break
INSIG1	insulin-induced gene 1
FASN	fatty acid synthase
TAG	triacylglycerol
MUFA	monounsaturated fatty acid
PUFA	polyunsaturated fatty acid
FA	fatty acid
FABP4	fatty acid-binding protein 4
QTL	quantitative trait locus
T2D	type-2 diabetes
sgRNA	single guide RNA
KO	knockout
siRNA	small interfering RNA
OT	off-target
SFA	saturated fatty acid
pri-miRNA	primary miRNA
pre-miRNA	precursor miRNA
BODIPY	Boron dipyrromethene
DAPI	4′,6-Diamidino-2′-phenylindole
