# Supplementary material for: CRISPR/Cas9-Induced Knockout of miR-24 Reduces Cholesterol and Monounsaturated Fatty Acid Content in Primary Goat Mammary Epithelial Cells
Source: Foods. 2022 Jul 7;11(14):2012. doi: 10.3390/foods11142012 (PMC9316712; doi:10.3390/foods11142012)
Supplement: Supplementary file 1 [file foods-11-02012-s001.zip › foods-1723507-supplementary.pdf]

Suppl. Table S1. The siRNA Sequences of *FASN* and *INSIG1*.

Name	Sense sequence (5′-3′)	Antisense sequence (3′-5′)
siNC	ACUACCGUUGUUAUAGGUGTT	CACCUAUAACAACGGUAGUTT
siFASN-369	GCAUCAACCCAGCUUCCAUTT	AUGGAAGCUGGGUUGAUGCTT
siFASN-4783	GCUCUUUGACAACCGGUAUTT	UGAGGGAGGCAUAAUAGAUTT
siFASN-5395	CCUGGAAAUUGGCAAAUUUTT	AAAUUUGCCAAUUUCCAGGTT
siINSIG1	GCCAAUAACGUUCAGCUCUTT	AGAGCUGAACGUUAUUGGCTT

Suppl. Table S2. Primers used for detection of sgRNAs in 11 off-target sites.

sgRNA	Off-target site	Primer sequence (5' to 3')	Size (bp)
sgRNA1	OT1	F: AGGAGCATAGGTTCGGTCATCGG<br>R: GCACAAGCCCTGGCACTTCGT	778
	OT2	F: GGTTTCCACTGGTTTGCCGTTAC<br>R: CCTTAGGGATCTTCTGATTCAGCATG	539
	OT3	F: CTCGGGCAGCGACTGACTCTTG<br>R: CTCGGGCAGCGACTGACTCTTG	1180
	OT4	F: GCGTGCCCAATGACAAGGTATGCG<br>R: CCCAACGCTGGAGGGTCGCTAA	1263
	OT5	F: CCTAATATCCACTCAGAGCCAATGCCATCA<br>R: CCAGAGGAGAAGAGCCCAGGACAACAG	1279
sgRNA2	OT1	F: GGGAGATTCAGGCTGCTTGGTTGG<br>R: CAGAGGACTGGCATGGGAAGTGGG	958
	OT2	F: CTGGGATTCTTTGTTCGTGTTGTTTG<br>R: GGGTCAGTTCTGAGTCGCTATTTCTTCT	458
	OT3	F: CAGAGGAGGGAGAAACCAGCGAATA<br>R: GGGCCGTGGCACGTACATCA	1051
	OT4	F: GGACTTGCACTCCCTCATCCTGTG<br>R: TTCTGTTGGTCTCATTCATTCGGTCA	1309
	OT5	F: CTCACATTTCCTCCTTCCAGTCTTCA<br>R: TGTAACCATCAGTCAATGCGTTATCC	1597
	OT6	F: TAGACTGCCCTCATTTCCCTGGTG<br>R: GTGCTTTGGAGATGGAGCTACGACTT	1133

Suppl. Table S3. Primers for reverse transcription quantitative PCR of miRNAs.

miRNA	Primer sequence (5' to 3')	Reference
miR-24-5p	Reverse: GTGCAGGGTCCGAGGTCAGAGCCACCTGGG CAATTTTTTTTTTTTGATATC<br>Forward: TTCGGGTGCCTACTGAGCT	This manuscript
miR-24-3p	Reverse: GTGCAGGGTCCGAGGTCAGAGCCACCTGGG CAATTTTTTTTTTTTGTTCCT<br>Forward: TTCGGTGGCTCAGTTCAGC	1
miR-23b-3p	Reverse: GTGCAGGGTCCGAGGTCAGAGCCACCTGGGCAATT TTTTTTTTTGGTAAT<br>Forward: TCGGATCACATTGCCAGGG	This manuscript
18S rRNA	Reverse: GTGCAGGGTCCGAGGTCAGAGCCACCTGGG CAATGCAGTGATGGCAAAGG<br>Forward: CAGCACATCTTGCGAGTACTC	2
Universal reverse primer	CAGTGCAGGGTCCGAGGT	1

Suppl. Table S4. Characteristics of primers for mRNA RT-qPCR.

NCBI accession no.	Gene	Primer sequence (5' to 3')	Size (bp)	Reference
JN236219.1	*ACACA*	F: CTCCAACCTCAACCACTACGG	171	3
		R: GGGGAATCACAGAAGCAGCC		
NM_001035289.2	*ACOX1*	F: CGAGTTCATTCTCAACAGTCCT	211	4
		R: GCATCTTCAAGTAGCCATTATCC		
BC119914	*ACSL1*	F: GTGGGCTCCTTTGAAGAACTGT	120	5
		F: ATAGATGCCTTTGACCTGTTCAAAT		
BC134532	*ACSS2*	F: GGCGAATGCCTCTACTGCTT	100	5
		R: GGCCAATCTTTTCTCTAATCTGCTT		
HQ846827	*ADFP*	F: TACGATGATACAGATGAATCCCAC	202	4
		R: CAGCATTGCGAAGCACAGAGT		
NM_00108366.1	*AGPAT6*	F: AAGCAAGTTGCCCATCCTCA	101	3
		R: AAACTGTGGCTCCAATTTCGA		
NM_001285739.1	*ATGL*	F: GGAGCTTATCCAGGCCAATG	180	6
		R: TGCGGGCAGATGTCACTCT		
FJ415874.1	*CPT1A*	F: AAGGACCTCTACGCCAACACG	267	4
		R: TTTGCGGTGGACGATGGAG		
DQ380249.1	*DGAT1*	F: CCACTGGGACCTGAGGTGTC	101	3
		R: GCATCACCACACACCAATTCA		
NM_001046597.1	*ELOVL6*	F: GGAAGCCTTTAGTGCTCTGGTC	205	7
		R: ATTGTATCTCCTAGTTCGGGTGC		
NM_174313.2	*FABP3*	F: GATGAGACCACGGCAGATG	120	7
		R: GTCAACTATTTCCCGCACAAG		
EE347846	*FADS1*	F: GGTGGACTTGGCCTGGATG	101	7
		R: TGACCATGAAGACAAGCCCC		
DV895683	*FADS2*	F: GCCTGTAGGCTCAGATGTTTGTTC	101	7
		R: TGCCTGGCAGTAACAGAGCAC		
DQ915966.3	*FASN*	F: GGGCTCCACCACCGTGTTCCA	226	3
		R: GCTCTGCTGGGCCTGCAGCTG		
AY515690	*GPAM*	F: ATTGACCCTTGGCACGATAG	188	5
		R: AACAGCACCTTCCCACAAAG		
EU273879	*HSL*	F: GGGAGCACTACAAACGCAACG	118	This manuscript
		R: TGAATGATCCGCTCAAACTCG		
NM_001286088.1	*INSIG1*	F: AGCCTCACAAGTTCAAGCG	132	8
		R: ACAGTGCTGCTAATGTCAAGG		
NM_001285738	*INSIG2*	F: ATACCCCTGCATTGACAGACAT	109	8
		R: ACCAAGGCCAAAACCACTTC		
XM_005681211.1	*PPARA*	F: CGGTGTCCACGCATGTGA	56	5
		R: TCAGCCGAATCGTTCTCCTAAA		
DT860044	*RPS9*	F: CCTCGACCAAGAGCTGAAG	64	9
		R: CCTCCAGACCTCACGTTTGTTC		

XM_005696121.1	*SCAP*	F: CCATGTGCACTTCAAGGAGGA<br>R: TGTCGATCTTGCGTGTGGAG	107	3
GU947654	*SCD1*	F: CCATCGCCTGTGGAGTCAC<br>R: GTCGGATAAATCTAGCGTAGCA	257	3
HM443643.1	*SREBP1a*	F: CTGCTGACCGACATAGAAGACAT<br>R: GTAGGGCGGGTCAAACAGG	81	This manuscript
HM443643.1	*SREBP1c*	F: CTGCTGACCGACATAGAAGACAT<br>R: GTAGGGCGGGTCAAACAGG	81	3
NC_030812.1	*SREBP2*	F: CGGCGTGATCGTGCTGAGCGTC<br>R: CCGACAGGCAGGTTTGCAGGTTGG	173	This manuscript
HQ846826	*TIP47*	F: GGTGGAGGGTCAGGAGAAA<br>R: TCACGGAACATGGCGAGT	123	4
NM_001037471	*UXT*	F: CAGCTGGCCAAATACCTTCAA<br>R: GTGTCTGGGACCACTGTGTCAA	125	9
BC102076	*XDH*	F: GATCATCCACTTTTCTGCCAATG<br>R: CCTCGTCTTGGTGCTTCCAA	100	This manuscript

ACACA, acetyl-coenzyme A carboxylase alpha; *ACOX1*, acyl coenzyme A oxidase 1; *ACSL1*, acyl-CoA synthetase long-chain family member 1; *ACSS2,* acyl-CoA Synthetase short-chain family member 2; *ADFP*, adipose differentiation-related protein; *AGPAT6*, 1-acylglycerol-3-phosphate O-acyltransferase 6; *ATGL*, adipose triglyceride lipase; *CD36*, rhrombospondin receptor; *C/EBPβ*, CCAAT enhancer binding protein beta; *CPT1A*, carnitine palmitoyltransferase 1A; *DGAT1*, diacylglycerol acyl transferase 1; *DGAT2*, diacylglycerol acyl transferase 2; *ELOVL6*, elongase of very long chain fatty acids 6; *FADS1*, fatty acid delta-6-desaturase 1; *FADS2*, fatty acid delta-6-desaturase 2; *FASN*, fatty acid synthase; *GPAM*, glycerol-3-phosphate acyltransferase; *HSL*, hormone-sensitive lipase; *INSIG1*, insulin-induced protein 1; *INSIG2*, insulin-induced protein 2; *PPARA*, peroxisome proliferator-activated receptor α; *RPS9*, ribosomal protein S9; *SCAP*, SREBP cleavage activating protein; *SCD1*, stearoyl-CoA desaturase 1; *SREBP1a*, sterol regulatory element-binding transcription protein 1a; *SREBP1c*, sterol regulatory element-binding transcription protein 1c; *SREBP2*, sterol regulatory element-binding transcription protein 2; *TIP47*, tail-interacting protein 47;

UXT, ubiquitously expressed transcript; *XDH*, xanthine dehydrogenase.

1. Wang, H.; Luo, J.; Chen, Z.; Cao, WT.; Xu, H. F.; Gou, D. M.; Zhu, J. J., MicroRNA-24 can control triacylglycerol synthesis in goat mammary epithelial cells by targeting the fatty acid synthase gene. *Journal of Dairy Science* **2015**, 98, (12), 9001-9014.

2. Chen, Z.; Luo, J.; Zhang, C.; Ma, Y.; Sun, S.; Zhang, T.; Loor, J. J., Mechanism of prolactin inhibition of miR-135b via methylation in goat mammary epithelial cells. *Journal of Cellular Physiology* **2018**, 233, (1), 651-662.

3. Shi, H.; Luo, J.; Zhu, J. J.; Li, J.; Sun, Y.; Lin, X.; Zhang, L.; Yao, D.; Shi, H., PPARγ regulates genes involved in triacylglycerol synthesis and secretion in mammary gland epithelial cells of dairy Ggoats. *PPAR Research* **2013**, *2013*, 310948.

4. Zhu, J. J.; Luo, J.; Wang, W.; Yu, K.; Wang, H. B.; Shi, H. B.; Sun, Y. T.; Lin, X. Z.; Li, J., Inhibition of FASN reduces the synthesis of medium-chain fatty acids in goat mammary gland. *Animal* **2014**, 8, (9), 1469-1478.

5. Bionaz, M.; Loor, J. J., Gene networks driving bovine milk fat synthesis during the lactation cycle. *BMC Genomics* **2008**, *9*, 366.

6. Lin, X.; Luo, J.; Zhang, L.; Wang, W.; Shi, H.; Zhu, J. J., miR-27a suppresses triglyceride accumulation and affects gene mRNA expression associated with fat metabolism in dairy goat mammary gland epithelial cells. *Gene* **2013,** 521, (1), 15-23.

7. Tian, H.; Luo, J.; Zhang, Z.; Wu, J.; Zhang, T.; Busato, S.; Huang, L.; Song, N.; Bionaz, M., CRISPR/Cas9-mediated stearoyl-CoA desaturase 1 (SCD1) deficiency affects fatty acid metabolism in goat mammary epithelial cells. *Journal of Agricultural and Food Chemistry* **2018,** 66, (38), 10041-10052.

8. Li, C.; Wang, M.; Zhang, T.; He, Q.; Shi, H.; Luo, J.; Loor, J. J., Insulin-induced gene 1 and 2 isoforms synergistically regulate triacylglycerol accumulation, lipid droplet formation, and lipogenic gene expression in goat mammary epithelial cells. *Journal of Dairy Science* **2019,** 102, (2), 1736-1746.

9. Bionaz, M.; Loor, J. J., Identification of reference genes for quantitative real-time PCR in the bovine mammary gland during the lactation cycle. *Physiological Genomics* **2007**, *29*, 312-319.

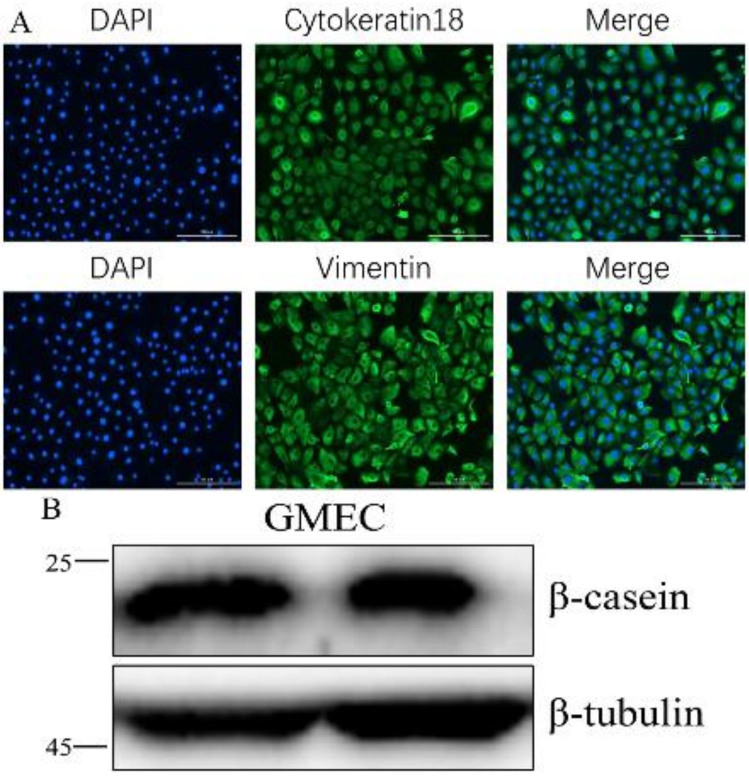

Suppl. Figure S1. Identification of primary goat mammary epithelial cells by immunofluorescence staining and western blot. (A) Immunofluorescence staining of Cytokeratin 18 and Vimentin in GMECs. Scale bar = 200 μm. (B) The expression of β-casein protein by western blot analysis in GMECs.

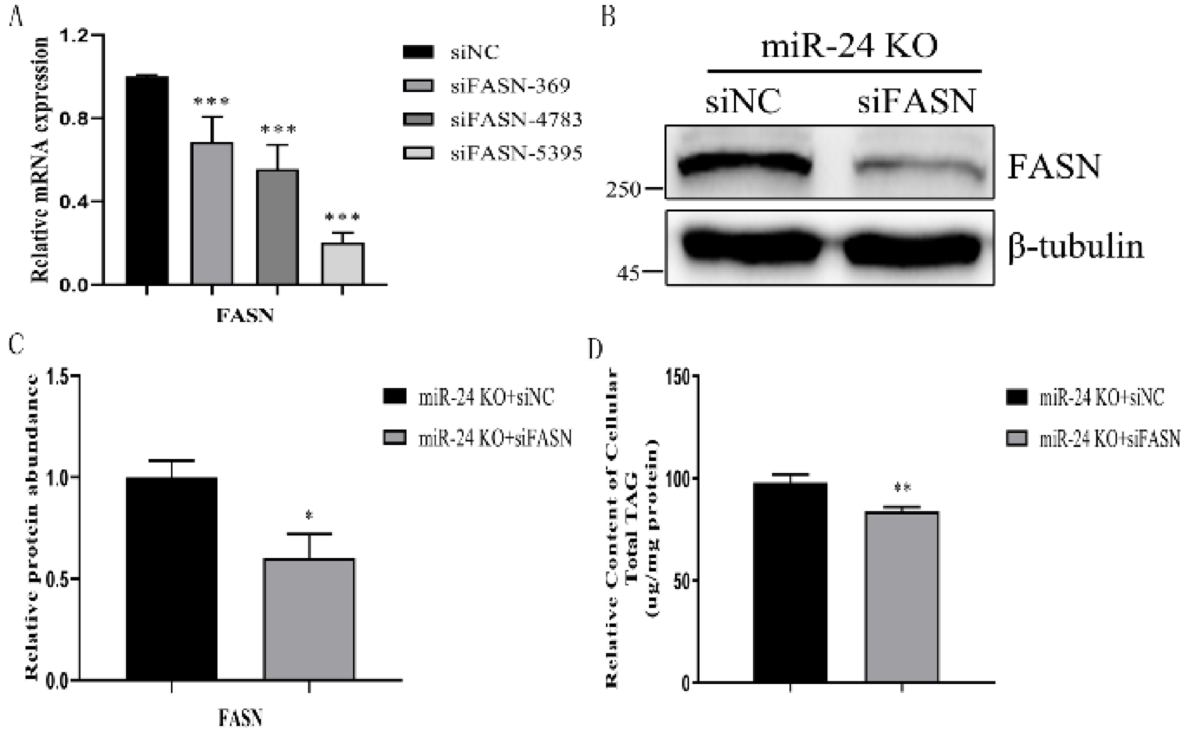

Suppl. Figure S2. Interference with *FASN* inhibits TAG synthesis in miR-24 knockout cells. (A) Interference efficiency verification of siFASN. (B, C) FASN protein expression analysis in miR-24 knockout cells transfected with siFASN or siNC. (D) The content of TAG in miR-24 knockout cells transfected with FASN siRNA or NC. * $p < 0.05$, ** $p < 0.01$, *** $p < 0.001$.

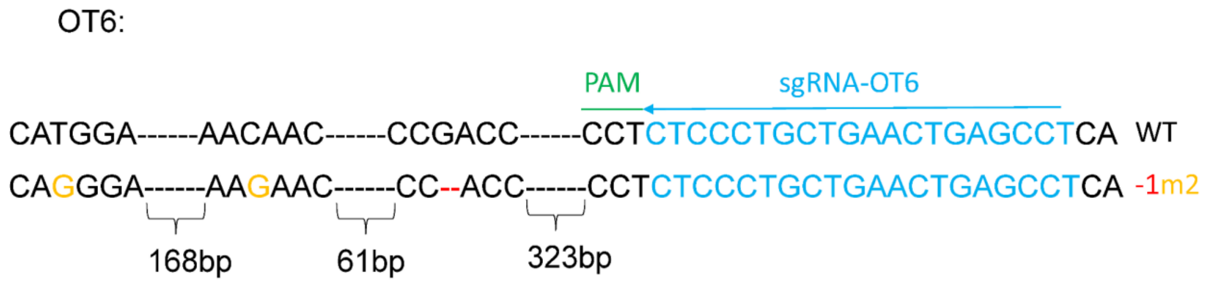

Suppl. Figure S3. Sequences of two alleles in off-target site 6 of sgRNA2.
